# R-28 cell-derived extracellular vesicles protect retinal ganglion cells in glaucoma

**DOI:** 10.4103/NRR.NRR-D-24-00709

**Published:** 2025-03-25

**Authors:** Esmahan Durmaz, Maryam Esmaeili, Philip Lewis, Gloria Cimaglia, Aled Clayton, Ben Mead

**Affiliations:** 1 School of Optometry and Vision Sciences, College of Biomedical and Life Sciences, Cardiff University, Cardiff, UK; 2 Dementia Research Institute, College of Biomedical and Life Sciences, Cardiff University, Cardiff, UK; 3 School of Medicine, College of Biomedical and Life Sciences, Cardiff University, Cardiff, UK

**Keywords:** extracellular vesicles, glaucoma, miRNA, neuroprotection, R-28 cell line, retinal ganglion cells

## Abstract

Glaucoma is characterized by chronic progressive optic nerve damage and retinal ganglion cell death. Although extensive research has been conducted on neuroprotection for retinal ganglion cells, there is still no treatment for clinical use. Recent evidence shows that extracellular vesicles isolated from a variety of stem cells are efficacious in retinal ganglion cell neuroprotection. In this study, we tested the novel extracellular vesicle source of the retinal progenitor R-28 cell line *in vitro* and *in vivo*. We isolated and characterized extracellular vesicles from R-28 cells and tested their therapeutic efficacy in terms of retinal ganglion cell survival *in vitro* and in an *in vivo* glaucoma model, measuring retinal ganglion cell survival and preservation of their axons. Additionally, we tested extracellular vesicles for their neuroprotective capacity in retinal ganglion cells differentiated from human embryonic stem cells. Finally, we investigated miRNA changes in retinal ganglion cells with R-28 extracellular vesicle treatment, and predicted possible pathways that may be modulated. R-28 extracellular vesicles improved retinal ganglion cell survival but failed to preserve axons significantly. Moreover, the results also illustrated the neuroprotection of R-28 extracellular vesicles on human retinal ganglion cells. Finally, we also showed changes in hsa-miRNA-4443, hsa-miRNA-216a-5p, hsa-let-7e-5p, hsa-miRNA-374b-5p, hsa-miRNA-331-3p, and hsa-miRNA-421 expressions, which may have neuroprotective potential on retinal ganglion cell degeneration. This study will pave the way for miRNA and extracellular vesicle-based neuroprotective therapies for glaucoma.

## Graphical abstract

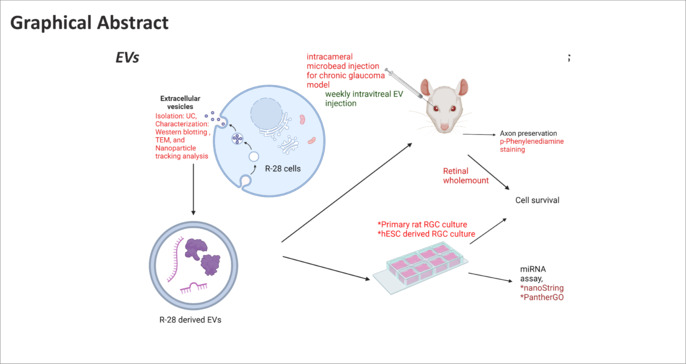

## Introduction

Extracellular vesicles (EV) are 50–150 nano-sized, lipid-bound natural carriers for cell-to-cell communication. EVs have emerged as powerful platforms for regenerative medicine with their capacity to deliver endogenous cargo to the target to promote tissue repair. EVs have been used in several preclinical studies for the treatment of different degenerative diseases including neurodegenerative and tissue injury. In retinal diseases, EVs have shown promise in neuroprotection against injuries resulting from various retinal diseases (Mead and Tomarev, 2017; Mathew et al., 2019; Seyedrazizadeh et al., 2020). For example, bone marrow mesenchymal stem cell (MSC)-derived EVs showed neuroprotective effects on retinal ganglion cells (RGC) after optic nerve crush via miRNA-dependent mechanism (Mead and Tomarev, 2017). Following this study, another group showed that human umbilical Wharton’s jelly-derived MSCs improved RGC survival up to 120 days after optic nerve crush (da Silva-Junior et al., 2021). Similarly, a recent publication showed that urine-derived EVs improved RGC survival, and endogenous miRNA-124 found within EVs from vitreous humor elicited neuroprotection of RGC (identified in retinal flat-mounts) and improved visual function (measured by electroretinography) after nonarteritic anterior ischemic optic neuropathy (Chen et al., 2023). EVs have also been isolated from MSC-derived from embryonic stem cells (ESC), which elicited RGC neuroprotection in a mouse model of optic nerve crush (Seyedrazizadeh et al., 2020). Alongside these studies, EV engineering, that is, the modification of EVs to enhance their effects, has also shown promising results for neuroprotection. EVs from cells exposed to hypoxic conditions showed more RGC protection and decreased inflammatory cytokine production via an increase in particular miRNAs (Mathew et al., 2023). To test the long-term effect of umbilical cord MSC-derived EVs, they were recently tested on animals with established chronic ocular hypertension, which were effective even after 7 weeks (Yu et al., 2023). In conclusion, studies have provided important information on the neuroprotective potential of EVs, however, it is still an emerging area and requires more research for clinical use, particularly as their therapeutic efficacy varies depending on their source. Isolating EVs from primary cells is also challenging, as the resulting EV product will vary depending on the passage of the cells, as well as their individual patient source. For clinical translation, it would be more ideal for therapeutic EVs to be isolated from a cell line to obtain more standardized product. This way, EVs will not suffer batch-to-batch variability and can be scaled up to meet patient demands, a critical requirement given the much higher dose that is likely needed to treat humans as opposed to rodents. The immortalization process itself is likely to change the cells and their secreted EVs however, so there is no guarantee an efficacious source will behave the same post-immortalization.

R-28 cells are an immortalized cell line from postnatal day 6 rat retinal culture, which has been used in various *in vivo* and *in vitro* studies (Seigel, 2014). Despite this extensive research on the R-28 cell line, there is no published research on EV studies from R-28 cells. In this study, R-28 cell line was used as a cell source for EV isolation as studies showed that EV natural tropism could be stemmed from the parent cell (Wiklander et al., 2015). Additionally, a study investigating microarray assays of R-28 cells revealed that these cells express genes for various neuroprotective proteins including insulin-like growth factor, platelet-derived growth factor, brain-derived neurotrophic factor, and transforming growth factor-beta (Seigel et al., 2004). Therefore, considering EVs are often a reflection of the cell membrane or cytoplasm content of the host cell, it may be possible that these proteins are carried by R-28 cells-derived EVs to recipient cells. In this study, we hypothesize that R-28 cell line could be used as an EV source and R-28-derived EVs could elicit neuroprotection on injured RGC, both in culture and in an *in vivo* model of glaucoma. We investigated the neuroprotective effect on RGCs *in vitro* in rat-derived primary RGCs and human ESC-derived RGCs. We then investigated *in vivo* RGC soma and axon protection in a rat glaucoma model induced by microbead injection into the anterior chamber of the eye. Finally, we investigated whether R-28 cell-derived EVs cause miRNA changes in RGCs and could explain their neuroprotective mechanism. Our results showed for the first time EVs obtained from R-28 cell line could be used as a therapeutic for RGC degeneration in glaucoma.

## Methods

### R-28 cell cultivation

R-28 cells were obtained from University of Rochester (RRID: CVCL_5I35) (Seigel, 2014). Cells were cultured with Dulbecco’s Modified Eagle Medium (Thermo Fisher Scientific, Waltham, MA, USA) containing 10% fetal bovine serum (FBS) (Sigma-Aldrich, Darmstadt, Germany), and 1% penicillin/streptomycin (Thermo Fisher Scientific). Media from cell culture at passages 9–15 was collected, pooled, and stored at –80°C until EV isolation. Before media collection for EV (**~**80% cell confluency), the medium was switched to an exosome-depleted FBS-contained medium (Thermo Fisher Scientific) and incubated for at least 24 hours.

### Extracellular vesicle isolation and characterization

**Figure 1:**
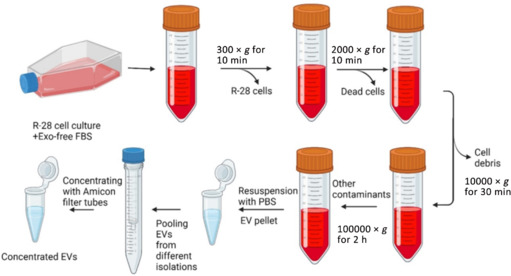
The process of extracellular vesicle isolation. EV: Extracellular vesicles; Exo: exosomes; FBS: fetal bovine serum; PBS: phosphate buffered saline.

Differential ultracentrifugation was used to isolate EVs as described in our previous study (Mead and Tomarev, 2017). Briefly, collected media was centrifuged at 300 × *g* for 10 minutes, the supernatant was then centrifugated at 2000 × *g* for 10 minutes, and finally this supernatant was filtered through a 0.22 µm filter and centrifugated at 10,000 × *g* for 30 minutes before a final 100,000 × *g* centrifugation of the supernatant for 2 hours. All centrifugation steps were performed at 4°C. After the last centrifugation, the pellet was resuspended in 500 µL DPBS and stored at –80°C until downstream processing. When concentrating EVs was necessary, Amicon filter tubes (Sigma) were used according to the manufacturer’s protocol. Briefly, 4 mL of EV sample was centrifugated at 4000 × *g* for 10 minutes, then resuspended with 500 μL DPBS and stored at –80°C until downstream processing (**Figure 1**).

For EV characterization and quantification, Western blotting, nanoparticle tracking analysis (NTA), and transmission electron microscopy (TEM) were used. EV size distribution analysis and particle count were performed by NTA (NanosightTM NS300 system, Malvern Analytical, Malvern, UK). EV protein markers were detected by Western blot. Briefly, cells/EVs were lysed in lysis buffer (20 mM Tris-HCl, 1 mM EDTA,0.5 mM EGTA, 150 mM NaCl, 1% NP-40, and protease inhibitor), protein concentrations determined by micro BCA assay before 1 µg protein samples were separated on 4%–12% Bis-Tris protein gels at 150 V for 40 minutes. Proteins were transferred to polyvinylidene fluoride membranes, blocked for 30 minutes in 10% Western blocking buffer in Tris-buffered saline (TBS), stained for 1 hour with CD81 primary antibody (**Table 1**) diluted in TBS, washed with TBST for 3 × 5 minutes, stained for 1 hour with secondary antibody before a final 3 × 5 minutes wash and detection with Femto ECL.

**Table 1:** The summary of antibodies used in this study

Antibody	Host	Supplier	Dilution	Cat#	RRID
βIII-Tubulin	Mouse	Sigma, Darmstadt, Germany	1:500	T8578	AB_1841228
Brn3a	Mouse	Millipore, Darmstadt, Germany	1:200	AB5945	AB_92154
CD81	Hamster	BioRad, Watford, UK	1:100	MCA1846	N/A
Alexa Fluor 488 for cell culture	Goat	Thermo Fisher Scientific, Waltham, MA, USA	1:400	A-11001	AB_2534069
Alexa Fluor 555 for retina wholemount	Goat	Thermo Fisher Scientific	1:500	A-21422	AB_2535844

Additionally, EV morphology was explored by TEM. Briefly, EVs and cells resuspended in serum-free media were gelled with 4% Ultra-low gelling temperature agarose (Sigma) at a temperature of 37°C and solidified in the fridge for 30 minutes at 4°C to fix in Karnovsky’s fixative (2.5% glutaraldehyde, 2% paraformaldehyde, 0.1 M Sorenson’s pH 7.2) for 3 hours. The pellets were cut and washed in Sorenson’s buffer three times over 30 minutes, post-fixed in 1% osmium tetroxide in the buffer for 1 hour, and washed with dH_2_O three times over 20 minutes before being transferred into 2% aqueous uranyl acetate (UA) and placed in a fridge overnight at 4°C. After washing with dH_2_O for 1 hour the samples were dehydrated in a 70%–100% ethanol series for 1 hour, placed in 100% Acetone for 30 minutes, and infiltrated with a 1:1 mix of 100% acetone and Araldite resin (Araldite monomer CY212 DDSA hardener and BDMA accelerator) for 2 hours. The samples were embedded and polymerized at 60°C for 48 hours. Ultrathin sections were cut (100 nm) using a Leica UC6 ultra-microtome (Leica, Wetzlar, Germany), these were collected on Agar 300 hexagonal TEM copper grids, stained with 1% UA in 70% Ethanol for 10 minutes, washed in 70% ethanol for 2 minutes and dH_2_O for 5 minutes. The EM grids were then imaged using a JEOL 1010 transmission electron microscope at an accelerating voltage of 80 kV, fitted with a Gatan Orius 1000 TEM camera (Gatan, Abingdon, England).

### miRNA isolation and NanoString assay

miRNA was isolated using Qiagen® miRNeasy® Advanced Micro kit (Qiagen, Hilden, Germany) as per the manufacturer’s instructions. The quality of RNA was assessed using a 2100 Bioanalyser (Agilent Technologies, CA, USA) and all samples had an RNA integrity number (RIN) value of 9.70–10. miRNA quantification using nCounter platform (NanoString, Seattle, WA, USA) involves several steps including miRNA sample preparation and miRNA CodeSet Hybridization setup. Briefly, oligonucleotide tags were annealed and then ligated with miRNA through a Bridge Oligo. Hybridization to the specific target of interest occurred using a Reporter CodeSet. The amount of RNA loading was 100 ng and Human v3 miRNA CodeSet kit (NanoSring) was used to set up miRNA CodeSet hybridization. The samples were hybridized on Veriti^TM^ Thermal Cycler (Applied Biosystems, Foster City, CA, USA) for 20 hours and then processed using the nCounter Prep Station (NanoString) and nCounter Digital Analyser (NanoString). The RNAseq data was analyzed using nSolver Analysis Software 4.0 (NanoString), whereby data was normalized to housekeeping genes. Since the assay has a minimum detection threshold for weakly expressed miRNA, it was necessary to filter the normalized abundance values. As such, when comparisons between two treatment groups were made, miRNA with abundance values of below 25 (in both groups) was removed. This applies to both the abundance measurements as well as the subsequent fold change heatmaps (whereby fold changes are presented as log2 fold change). Heatmaps further filtered out any average fold change that was not > 2 or ≤ 2. Nanostring mRNA targets were predicted using Ingenuity Pathway Analysis (IPA) software (Qiagen). The genes obtained from IPA were transferred to PantherGO (http://pantherdb.org) for further analysis. Results were visualized in Excel (Microsoft, Redmond, WA, USA).

### *In vitro* testing of extracellular vesicles on primary retinal cell culture

Adult female albino (Sprague-Dawley, 2–4 months) rats (**~**200 g, purchased from Charles River Laboratories, Cambridge, UK) were euthanized by CO_2_. We only used female rats to reduce costs, animal numbers, and as little evidence exists for sex having an effect in this glaucoma model. All rats were accommodated in cages with 1 to 5 animals. Room temperature and humidity were controlled at 20–24°C, and 55% ± 10%, on a 12-hour light/dark cycle, from 06:00 to 18:00. All animal work was performed under the Home Office Project License PP8112408. Primary retinal cultures were performed as we have previously described (Lorber et al., 2002; Mead and Tomarev, 2017; Durmaz et al., 2023). Briefly, the eyes were removed and placed in neurobasal-A (Gibco) on ice. The 8-well cell culture chamber slides (Merck Millipore, MA, USA) were prepared with 300 µL poly-D-lysine (Sigma, 100 µg/mL) for 60 minutes and 300 µL laminin (Gibco, natural mouse, Cat# 23017-015, 20 µg/mL) for 30 minutes. The dissected and minced retina was incubated in 1.25 mL papain (Worthington Biochemical, Lakewood, NJ, USA) and 62.5 μL DNase I (Worthington Biochemical) for 90 minutes at 37°C. After 90 minutes, cells isolated from the retina were centrifuged at 300 × *g* for 5 minutes. Subsequently, the pellet was resuspended in 1.35 mL of Earle’s Balanced Salt Solution (Worthington Biochemical), 150 µL of reconstituted albumin ovomucoid inhibitor (Worthington Biochemical), and 75 µL DNase I. To form a discontinuous density gradient to isolate cells, the albumin ovomucoid solution was centrifuged at 70 × *g* for 6 minutes. The pellet was resuspended with supplemented neurobasal-A. The cell pellet and neurobasal-A supplemented with 500 µL B27 (Gibco), L-Glutamine (200 mM) (Thermo Fisher Scientific), and 125 µL gentamycin (Thermo Fisher Scientific) mixture was added to the wells that were prepared as 125,000 cells per 300 µL (300 µL per well). Treatments included R-28-derived EVs, ciliary neurotrophic factor (positive control), and no treatment.

### *In vitro* testing of R-28 cell-derived extracellular vesicles on human embryonic stem cell-derived retinal ganglion cells

To test the therapeutic effect of R-28-derived EVs on human ESC-derived RGC (H7/H9 immortalized cell line; WiCell, Madison, WI, USA, #WA07, RRID: CVCL_S800) were differentiated from CRISPR-modified ESC generously donated from Prof Donald Zacks laboratory (Johns Hopkins University, Baltimore, MD, USA) and licensed for use from WiCell (Material Transfer Agreement issue-164634007). Differentiation was performed through the addition of small molecules, as has already been described (Sluch et al., 2017; Esmaeili and Mead, 2023). To maintain the cells, iNS media containing DMEM/F12 and neurobasal (50:50), GlutaMax 1×, antibiotic antimitotic 1×, N2 1×, and vitamin B27 1× was used. ESC-derived RGCs were treated with 2.5 × 10^9^ particles EVs with injury-induced using 1 μM colchicine incubated for 48 hours at 37°C.

### Immunocytochemistry

In this study, RGCs are defined as having a positive staining for βIII-tubulin and DAPI, with primary RGCs displaying preferential βIII-tubulin staining on one side of the cell. Cells (rodent retinal cultures and human RGC) were fixed with 4% paraformaldehyde (Sigma) for 10 minutes, followed by three times PBS wash for 10 minutes. The wells were treated with a blocking buffer that includes 3% bovine serum albumin (Sigma) and 0.1% Triton X-100 (Sigma). The primary antibody (monoclonal βIII-tubulin; dilution factor 1:500, Sigma, as shown in **Table 1**) was prepared with antibody dilution buffer containing 3% BSA and 0.05% tween 20 (Sigma) in PBS. The wells were incubated at room temperature for 1 hour with primary antibody, followed by three times PBS wash each for 10 minutes. The secondary antibody (goat anti-mouse IgG Alexa Fluor 488, dilution 1:400, Thermo Fisher Scientific, as shown in **Table 1**) was prepared in antibody dilution solution and each well incubated with 150 μL for 1 hour at room temperature in the dark, followed with three PBS washes. Lastly, the cell culture chamber slide was counterstained with DAPI (Vectashield, Newark, NJ, USA). The visualization of immunocytochemistry labeling was performed with a Leica AF6000/MBF system (Danaher Corporation) and LAS X (Danaher Corporation, Version 3.0.4.16529) imaging software.

### *In vivo* assessment of R-28 cell-derived extracellular vesicles

#### In vivo experimental design

All experiments were performed under the United Kingdom Animals (Scientific Procedures) Act 1986 after approval from the Home Office (PP8112408; approved September 2020) and the local Animal Welfare and Ethical Review Body (AWERB) committee. Room temperature and humidity were controlled at 20–24°C, and 55% ± 10%, on a 12-hour light/dark cycle, from 06:00 to 18:00. Rats (as detailed above) were randomly separated into three groups as shown in **Table 2**: Group 1 consisted of 5 intact animals; Group 2 consisted of microbead + PBS injected 3 rats; and finally, Group 3 consisted of microbead and R-28 derived EV injected 3 rats. Microbead injection was performed bilaterally into the anterior chamber to induce ocular hypertension as detailed below. The first EV injection was carried out on day 7 after intraocular pressure (IOP) elevation, also bilaterally. IOP and animal weight measurements were performed regularly at the same time on the day. At the end of the experiment, animals were euthanized with CO_2_, and eyes/optic nerves were collected for further analysis.

**Table 2:** *In vivo* R-28 cell-derived extracellular vesicle therapeutic testing experimental plan

Group	Number of animals	Microbead injection	Treatment
1	5	No injection	No treatment
2	3	Bilateral microbead injection	R-28 cell-derived extracellular vesicles (2.5 × 10^9^ particles)
3	3	Bilateral microbead injection	PBS (5 μL)

### Induction of ocular hypertension

Ocular hypertension induction was performed by blocking the outflow of aqueous humor according to previously published protocols (Weber and Zelenak, 2001; Urcola et al., 2006; Ito et al., 2016) with modification magnetic microbeads (Invitrogen, Carlsbad, CA, USA) (Samsel et al., 2011). Briefly, we used 5 µL of magnetic microbeads (2.7 × 10^6^ microbeads/µL) with their epoxy groups removed through treatment with 0.02 M NaOH in Tris Buffer, and sterilized with 0.4% oxybuprocaine hydrochloride and 0.5% chloramphenicol. Adult female Sprague-Dawley rats (Charles River, UK; 200–350 g) were anesthetized with 5% isoflurane inhalation (TEVA, Eastbourne, UK) in medical oxygen at a flow rate of 2 L/min and maintained at 2.5% isoflurane. The intracameral injection was performed with a 10 µL Hamilton syringe and 5 µL of microbeads were injected to induce glaucoma, and gradual neuron loss. Lubricant gel was applied to the animal to avoid dry eyes. A small magnet was used to move the injected beads into the iridocorneal angle.

IOP measurement was performed with a tonolab tonometer (Icare, Helsinki, Finland) under anesthesia, and IOP was measured twice a week three times for each eye (each measurement an average of 6 for a total of 18 measurements). After IOP measurement, animal weight was recorded and lubricant gel was applied to each eye.

### Intravitreal injection of R-28 cell-derived extracellular vesicles

The needles (self-made disposable sterile glass micropipettes [Harvard Apparatus, Kent, UK]) were prepared with 5 µL volume containing 2.5 × 10^9^ R-28-derived EV particles. The needle was injected into the vitreous through the sclera at 45° angle to avoid the lens and retina under anesthesia. Before putting the animal into the cage, lubricant was applied to the eyes.

### Retina wholemount immunostaining for retinal ganglion cell counts

Labeling of retinas in wholemount was performed as previously described (Miralles de Imperial-Ollero et al., 2023). Briefly, fixed retinas from SD rats were permeabilized with 0.5% triton for 10 minutes twice at room temperature before being frozen at –70°C for 15 minutes (in triton) and left to thaw for 1 hour at room temperature. They were then washed twice with 0.5% triton and incubated overnight at 4°C with primary antibody (Brn3a as shown in **Table 1**) in 2% normal goat serum/2% triton in PBS. The following day, retinas were washed with 0.5 % triton four times for 10 minutes at room temperature and incubated with secondary antibody (Alexa Fluor 555 as shown in **Table 1**) for 2 hours at room temperature. Retinas were washed with 0.5% triton twice and PBS and mounted vitreous side up in anti-fade mounting medium. Retinal whole mounts were visualized using Leica AF6000/MBF systems microscope camera DFC350 FX. Labeled RGCs were counted in three different areas from the optic nerve at three distances. Counts were performed manually. The mean number of RGC/image was derived from the 12 images (0.04 mm^2^) from each group consisting of retinas from 11 rats.

### Cryosectioning optic nerve and paraphenylenediamine staining

After 4-week post-initial injection, the animals were culled, and the optic nerves were removed immediately. Optic nerves were placed in 4% paraformaldehyde in PBS for 6 hours followed by 10%, 20%, and 30% sucrose (Alfa Aesar, Haverhill, MA, USA) in PBS for 8 hours each. After sucrose treatment, optic nerves were placed into cryomolds with an optimal cutting temperature medium. Fifteen sections from each optic nerve were cut with a cryostat (Leica, CM3050 S).

After cryosectioning, optic nerve sections were stained with 1% paraphenylenediamine (PPD) in isopropanol/methanol 1:1 for 45 minutes and mounted with permount mounting media (Thermo Fisher Scientific). Three investigators evaluated the results, scoring as mild, moderate, or severe, as previously described (Libby et al., 2005a).

### Statistical analysis

All statistics were conducted on GraphPad Prism 9.3.0 for Windows (GraphPad Software, Boston, MA, USA, www.graphpad.com). All data is shown as mean ± standard error of the mean and normality was tested using Shapiro-Wilkes test before parametric tests. To compare two groups, a ratio paired or unpaired *t*-test was performed. For the *in vivo* models with smaller sample sizes, Kruskal-Wallis test was used. NanoString miRNA data was analyzed using analysis of variance with Tukey’s *post hoc* test. Statistical differences were considered significant at *P* values < 0.05.

## Results

### R-28 cells secrete extracellular vesicles with general extracellular vesicle markers

**Figure 2:**
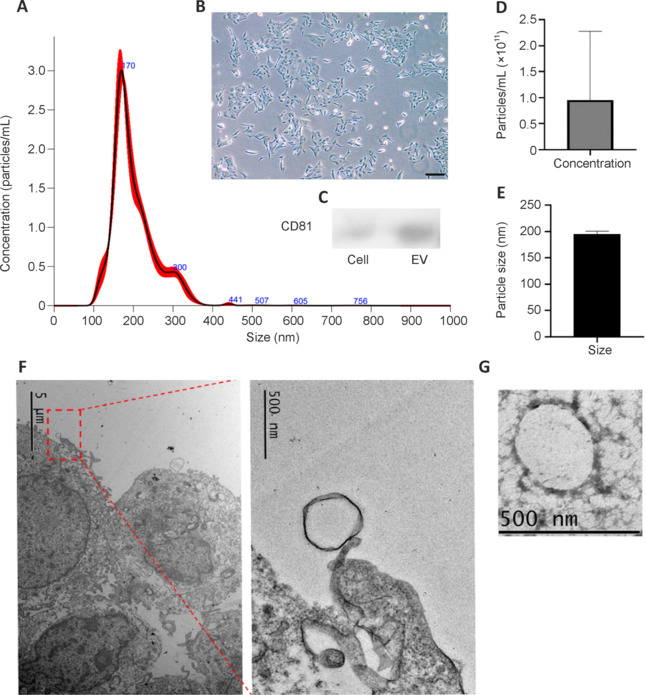
The diameter and concentration of particles present in EV preparation were determined by nanoparticle analysis and immunoblotting with EV-specific markers. EV concentration by particle diameter was obtained from Nanosight (A) after isolation from R-28 cells shown as magnified at 10×, Scale bar: 50 µm (B). EVs were enriched for the EV marker CD81 compared to R-28 cell lysates when the same density of protein was loaded (C). Over three separate isolations, the average concentration (D) and size of the EVs (E) were determined by Nanosight (*n* = 3). Electron microscopic imaging of the R-28 cells with EVs on the cell surface is visible (inset; F), and EVs were also detected in the purified EV preparation (G). EV: Extracellular vesicle.

EVs were isolated from R-28 cell line (Seigel, 2014) which showed a classic elongated spindle shape (**Figure 2B**). Nanoparticle tracking analysis revealed that the nanoparticle size in R-28 cell-derived EV preparation was relatively homogenous with sizes ranging from 100 and 400 nm. The mean particle size was determined as 200.8 ± 1.1 nm and the mode was 171.2 ± 3.3 nm (**Figure 2E**). The average concentration/yield was 1 × 10^11^ particles in 1:100 diluted EV solution, a typical isolation from 180 ml media (assuming 15 mL of media per 6 × 10^6^ cells in a T75 flask; **Figure 2A** and **D**).

Immunoblotting displayed higher expression of EV phenotypic marker CD81 in EVs, with relatively less protein expression in cell lysate. Cell lysates serve as a useful negative control as within the same quantity of protein, they will have significantly less CD81, which is expected to be enriched in samples with purified EVs (Abello et al., 2019; Arteaga-Blanco et al., 2020; **Figure 2C**). Transmission electron microscopy imaging of the R28 cells (**Figure 2F**) and of the purified EV preparation (**Figure 2G**) revealed the presence of nanoparticles of the expected size. These results suggest that EVs were secreted from R-28 cell line with proper characteristics.

EV characterizations and quantifications were performed routinely on every sample, and thus, while differences in yield were common, the delivered treatments were standardized based on these quantifications.

### *In vitro* assessment of neuroprotective/neurodegenerative potential of R-28 cell-derived extracellular vesicles

**Figure 3:**
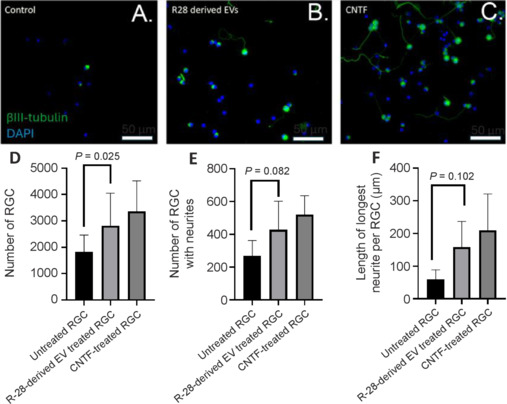
*In vitro* testing of R-28 cell-derived EVs on RGC survival and regeneration. Representative images of untreated control wells (A), R-28 cell-derived EVs treated wells (B), and CNTF-treated wells (C) are shown with the graphs showing the total surviving RGC number (D), the number of RGC with neurites (E), and the longest neurite length (F) in primary retinal cell culture after 3 days. Data are expressed as the mean ± SEM. Images were stained with a nuclear (DAPI, blue) and RGC marker (β-III tubulin, green). Scale bars: 50 µm. All experiments were performed in three independent biological replicates. CNTF: Ciliary neurotrophic factor; DAPI: 4′,6-diamidino-2-phenylindole; EV: extracellular vesicles; RGC: retinal ganglion cells.

We investigated the neuroprotective effect of R-28-derived EVs (2.5 × 10^9^ particles) in primary retinal cell culture (**Figure 3A–C**). The treatment showed significantly improved RGC survival (2814.33 ± 712.59 RGCs/well) compared to untreated control wells (1827 ± 368.38 RGCs/well, *P* = 0.025), in which 35.08% more RGC were detected in EV-treated wells (**Figure 3D**). We also investigated the neuritogenesis effect of R-28 cell-derived EVs, which proved to be similarly regenerative to that of CNTF treatment, with the number of RGCs with regenerating neurites in R-28 cell-derived EVs treated wells approaching significance compared to untreated wells (*P* = 0.082; **Figure 3E**). Moreover, we also compared the length of the neurites, which showed a similar pattern to the number of neurons with neurites. The neurite length of R-28 cell-derived EVs treated RGCs was on average, longer than the control group (58.6 9 ± 17.36 μm and 157.8 ± 45.86 μm, *P* = 0.102 respectively; **Figure 3F**) and was similar to CNTF treated RGCs, again approaching significance. Overall, these results indicate that R-28 cell-derived EVs protected RGCs from degeneration.

### Testing of neuroprotection in human embryonic stem cell differentiated retinal ganglion cells

**Figure 4:**
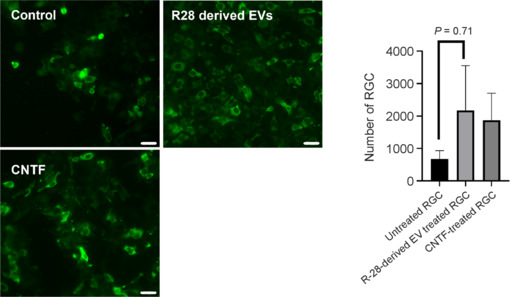
R-28 cell-derived EVs promote human ESC-derived RGC survival *in vitro*. Images show untreated controls, R-28 cell-derived EV treated, and CNTF-treated hESC-derived RGCs (green, βIII-tubulin) after injury induced by the microtubule poison, colchicine. Scale bar: 50 µm. Data are presented as mean ± SEM. All experiments were performed in three independent biological replicates. CNTF: Ciliary neurotrophic factor; ESC: embryonic stem cells; EV: extracellular vesicles; RGC: retinal ganglion cells.

R-28 cell line is derived from rat cells (Seigel, 2014), and their efficacy on rat retinal cells may not be recapitulated when used on human RGCs or may even show negative effects. Therefore, we investigated EVs on human RGCs differentiated from human ESCs to detect possible differences. R-28 cell-derived EVs are neuroprotective on hESC-derived RGCs (injured with colchicine) compared to untreated hESC-derived RGCs injured with colchicine (2178.67 ± 795.43 RGCs/well, 682.33 ± 145.89 RGCs/well, respectively, *n* = 3, *P* = 0.071) (**Figure 4**). Interestingly, these EVs trended to be more neuroprotective than CNTF with 16.65% more surviving RGCs. In summary, these results indicate that R-28 cell-derived EVs are neuroprotective even in human-derived RGCs and did not show any apparent neurotoxic effects.

### *In vivo* assessment of neuroprotective potential of R-28 cell-derived extracellular vesicles

**Figure 5:**
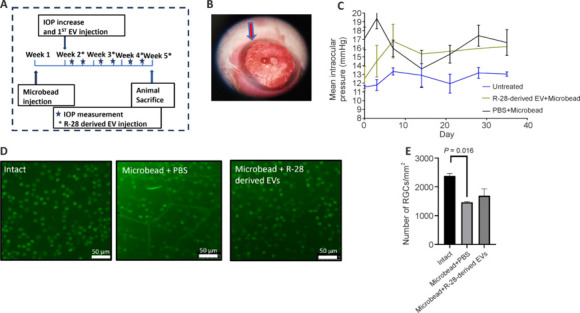
R-28 cell-derived EVs show protective trend for RGCs in a chronic glaucoma model. (A) Experimental design of the *in vivo* study. R-28 cell-derived EVs were intravitreally injected weekly beginning 1 week after microbead injection, and animals’ IOPs were measured twice a week. Four weeks after weekly EV injection, animals were sacrificed and histologically analyzed. After injection, microbeads localized (arrow) around the iridocorneal angle (B). IOP (mmHg) of healthy animals (blue) and animals receiving intracameral injection of microbeads with (green) or without (brown) intravitreal EV treatments is shown (C). (D, E) Representative images (D) and quantification (E) of Brn3a^+^ (green) RGCs from the three groups on week 5. Scale bars: 50 µm. Data are presented as mean ± SEM. *n* = 3–5. EV: Extracellular vesicles; IOP: intraocular pressure; RGC: retinal ganglion cells; PBS: phosphate buffered saline.

We investigated the effects of R-28 cell-derived EVs after weekly intravitreal injection into an *in vivo* rat model of glaucoma (**Figure 5A**). Microbead intracameral injection (**Figure 5B**) increased IOP to 17.1 ± 1.00 mmHg on day 7 (**Figure 5C**) whereas IOP remained low in the control group (11.57 ± 0.16 mmHg). The increased IOP was sustained until the end of the experiment (day 36: 16.17 ± 2.20 mmHg).

The number of Brn3a^+^ RGCs after microbead injection was significantly decreased (1463 ± 58.52 RGCs/mm^2^) compared to the intact group (2383 ± 80.725 RGCs/mm^2^, *P* = 0.002; **Figure 5D** and **E**). Weekly injection of 2.5 × 10^9^ R-28 cell-derived EV elicited some level of neuroprotection on Brna3a^+^ RGCs (1690.25 ± 239.4 RGCs/mm^2^, *P* = 0.3991). Together these results provide insights into the neuroprotective trend of R-28 cell-derived EVs *in vivo*.

### R-28 cell-derived extracellular vesicles are not protective for axons of R-28 cell-derived extracellular vesicles treated retinal ganglion cells *in vivo*

**Figure 6:**
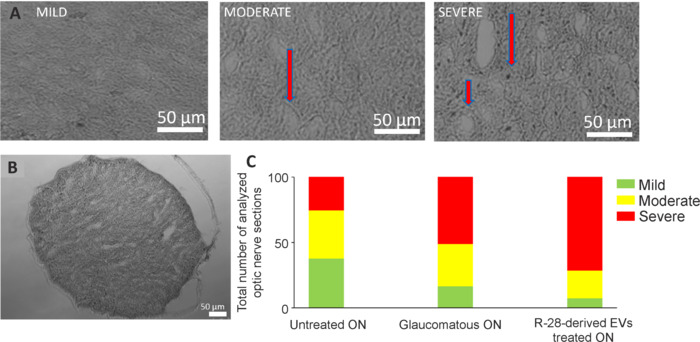
Optic nerve damage in glaucomatous eyes after EV treatment. (A) The representative images from different scales of damage, mild, moderate, and severe classified optic nerve sections. Red arrows show gliosis. Scale bars: 50 µm. (B) An example of a paraphenylenediamine-stained optic nerve section. The frequency of damage is shown as the percentage of the total number of analyzed optic nerve sections (C; *n* = 5). EV: Extracellular vesicles.

To investigate if R-28 cell-derived EVs preserve RGC axons in the optic nerve, we stained optic nerve sections with PPD staining, and scored damage on the optic nerve as mild, moderate, or severe (**Figure 6A** and **B**). The percentage of axons that received a severe grading was higher in the glaucomatous optic nerve group (16.21%, 32.43%, and 51.35%, respectively), compared to the uninjured group (37.59%, 36.84%, and 25.56% respectively; **Figure 6C**) but no difference was observed for the R-28 cell-derived EV treated group. In summary, these results suggest that R-28 cell-derived EVs failed to protect the ON.

### miRNA changes in R-28 cell-derived extracellular vesicles treated human retinal ganglion cells

**Figure 7:**
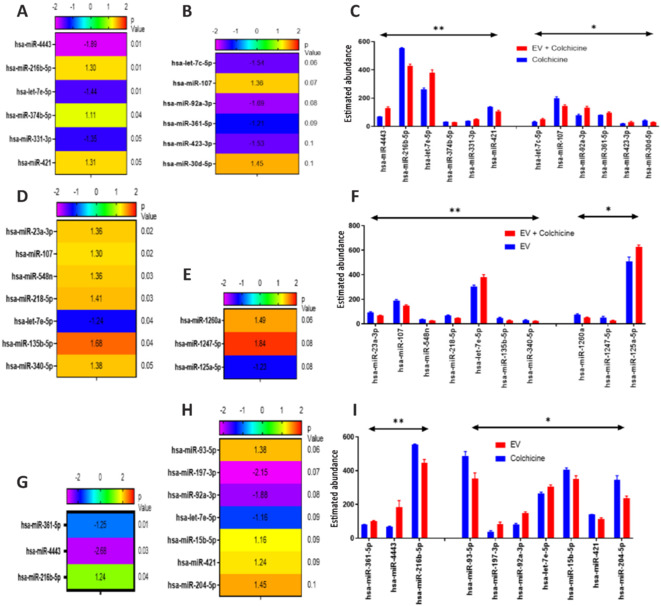
Differentially expressed miRNA shown as abundance and fold change heat map profiles. Heatmaps show the upregulated and downregulated normalized counts of miRNA from injured RGCs treated with R-28 cell-derived EVs compared to injured untreated (A, B), injured RGCs treated with R-28 derived EVs compared to uninjured treated (D, E), and uninjured RGCs treated with R-28 cell-derived EVs compared to injured untreated (G, H), both statistically significant (*P* < 0.05; A, D, G) and those trending towards significance (*P* < 0.1; B, E, H) with abundance profiles shown in associated bar charts (C, F, I, respectively). *P < 0.05, **P < 0.01. Data are presented as mean ± SEM. *n* = 3. EV: Extracellular vesicles; RGC: retinal ganglion cells.

After a neuroprotective effect was established, we performed miRNA nCounter identification assay on EV-treated RGCs (after injury with colchicine) to investigate genes and pathways that might be involved in the R-28 EV-mediated neuroprotective effect in the human RGC colchicine injury model. Data from NanoString Ncounter Technology shows that 6 of these miRNAs were changed significantly (*P* < 0.05). We found that the expression of hsa-miRNA 4443, hsa-let-7e-5p, and hsa-miRNA-331-3p were significantly higher in EV-treated RGCs after colchicine injury (*P* < 0.05; **Figure 7A** and **C**). In contrast, the expression of hsa-miRNA-216b-5p, hsa-miRNA-421 and hsa-miRNA-374b-5p was reduced in EV-treated RGCs and other, less pronounced differences were observed including hsa-let-7c-5p, hsa-miRNA-107, hsa-miRNA 92a-3p, hsa-miRNA-361-5p, hsa-miRNA-423-3p, and hsa-miRNA-30d-5p. Several other miRNA differences were observed that approached significance (**Figure 7B**). Alongside this comparison, we also compared EV^+^Colchicine^+^ RGC miRNA with and EV^+^Colchicine^–^ RGC miRNA (**Figure 7D–F**) as well as EV^+^Colchicine^–^ RGC miRNA with EV^–^Colchicine^+^ RGC miRNA (**Figure 7G–I**). According to our results, EV treatment significantly increased hsa-miRNA-4443, hsa-miRNA-216b-5p, and hsa-miRNA-let-7e-5p.

**Figure 8:**
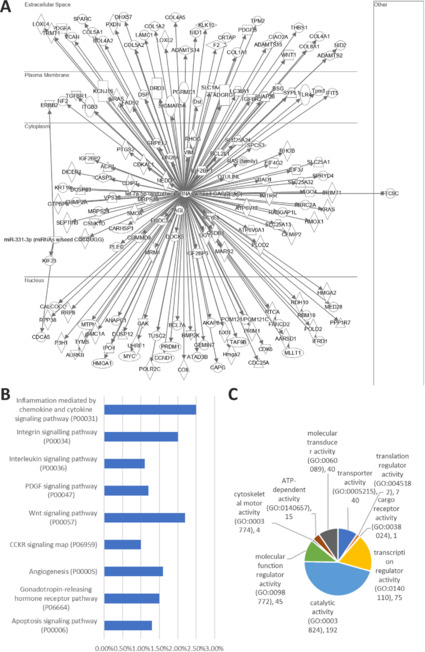
Schematic illustration of miRNA-experimentally and highly predicted mRNA targets. The three miRNAs significantly changed by R-28 cell-derived EV intervention were analyzed using IPA (243 genes). For further analyses, experimentally and highly predicted miRNA genes were selected and analyzed using PantherGO. Targeted genes were detected with IPA software (A) as well as the functional classification of the targeted pathways (B) and the associated biological processes (C). EV: Extracellular vesicles; IPA: ingenuity pathway analysis.

To predict genes and pathways possibly modulated by these candidate miRNAs, we performed *in silico* IPA gene target prediction and gene enrichment analysis, focussing only on “experimentally observed” candidates. The gene targets predicted by IPA are located in the nucleus, cytoplasm, plasma membrane, and extracellular space (**Figure 8A**). These miRNAs (hsa-let-7e-5p and hsa-miRNA-331-3p) were predicted to target 157 and 3 genes, respectively. The predicted targets of the differentially expressed miRNAs were analyzed using PantherGO for the top 10 most relevant pathways. These pathways include immune cell activation, platelet-derived growth factor signaling pathway (P00047), Wnt signaling pathway (P00057), and apoptosis signaling pathways (P00006) (**Figure 8B**). Further analysis showed the most targets related to catalytic activity (**Figure 8C**).

## Discussion

EVs have emerged as powerful platforms for regenerative medicine due to their capacity to deliver endogenous cargo to the target to promote tissue repair. This study showed that R-28 cell-derived EVs might be a potential treatment for the RGC degeneration associated with glaucoma, supported by *in vitro* and *in vivo* RGC survival testing. However, R-28 cell-derived EVs did not show a protective effect on optic nerve damage stimulated by microbead-mediated IOP increase, suggesting their effects are principally on the RGC soma. Moreover, we showed R-28 cell-derived EVs significantly changed the expression of miRNA 374b-5p, 331-3p, 421, 4443, 216b-5p, and let-7e-5p in degenerating RGCs.

EVs are nanoparticles secreted by cells that act as communicative signals via their miRNAs, mRNA, and protein cargo. In this study, we successfully isolated EVs via differential ultracentrifugation and obtained the appropriately sized CD81^+^ EVs. In recent studies, cell transplantation showed promising results in reducing RGC loss in glaucoma (Mead et al., 2013). For example, BMSC and dental pulp stem cell injections after ONC improve RGC survival (Mead et al., 2013). However, the difficulties of cell therapies limit the use of them in clinics. Therefore, EVs from cells may provide therapeutic effects without the undesired effects of cell therapies such as unwanted differentiation and/or retinal detachment. EVs have been investigated in different glaucoma models and shown improved RGC survival (Mead and Tomarev, 2017; Mead et al., 2018). These are similar to our own findings in rat primary RGC culture, showing that R-28 cell-derived EVs increase the number of surviving RGCs, although the effect appears less significant than seen with EVs from some stem cell courses. The neuritogenesis effect of EVs was similar to effects seen from various other sources including ESC-derived MSCs, and Schwann cells (Seyedrazizadeh et al., 2020; Zhu et al., 2023).

Microarray assays of R-28 cells have revealed the presence of various neuroprotective proteins including brain-derived neurotrophic factor, fibroblast growth factor, and transforming growth factor-β2 (Seigel et al., 2004). Considering EVs are often a reflection of the cell membrane or cytoplasm content of the host cell, speculatively these proteins may also be carried by R-28 cell-derived EVs, suggesting protein analysis of R-28 cell-derived EVs may be warranted in the future. This therapeutic cargo may cause toxic effects on human RGC that are not seen in rat retinal cultures (Guo et al., 2014; Weinreb et al., 2018). Our present findings however demonstrate that R-28 cell-derived EVs, originating from rats, improved survival of both human and rat RGCs and this neuroprotective effect was close in efficacy to our positive control, CNTF. CNTF is a potent neuroprotective, but its efficacy is limited to *in vitro* studies where it is not cleared, and delivery problems do not hamper its success.

Microbead occlusion creates elevated IOP by blocking aqueous outflow from the anterior chamber (Mead, 2023). In the present study, we elicited elevated IOP that was sustained for 4 weeks and led to RGC and optic nerve degeneration. RGC numbers in intact rats were similar to those reported in various studies (Kwong et al., 2011; Rodriguez et al., 2014; Mead et al., 2018) and microbead injection stimulated expected numbers of RGC loss (Mead et al., 2018). Weekly intravitreal R-28 cell-derived EV injection elicited neuroprotective trend, however, it is unknown if these EVs could provide sustained neuroprotection without the need for weekly injection.

Despite successful protection of RGCs *in vitro* and *in vivo*, R-28 cell-derived EVs failed to prevent ocular hypertension-induced axonal degeneration. Umbilical cord MSCs, which elicited glial inhibition and RGC protection in the retina after intravitreal transplantation however similarly failed to improve optic nerve preservation (Pan et al., 2019). The subjective way optic nerves are graded in this study did however lead to considerable variation between investigations with some nerves graded as severely damaged even in the healthy nerves. Different mechanisms are activated in RGC soma and axons in elevated IOP and it has been shown that axonal degeneration starts before RGC loss (Buckingham et al., 2008). Similarly, the deletion of BAX also reduces RGC loss, but not axonal loss in DBA/2J mice (Libby et al., 2005b). Therefore, R-28 cell-derived EVs might only target pathways for RGC soma loss, not axonal degeneration.

As a further analysis, we performed miRNA identification assay to see if there was a change in injured RGCs after EV treatment. Our results showed that one of the mechanisms by which R-28 cell-derived EVs may exert their effect through delivery or modulation of candidate miRNA. Our miRNA target prediction showed that key target pathways may be related to immune cells and thus acting through a microglial/astrocyte axis (Shinozaki et al., 2023), although this is only applicable to the *in vivo* model, and thus cannot be the exclusive mechanism of action. R-28 cells display a surprising amount of heterogenicity for a cell line, with high expression of glial markers compared to many retinal neurons (Seigel et al., 1996, 2018), possibly suggesting these EVs may target and exert their actions through retinal glia. Some of the candidate miRNA have been described to have roles in neurodegeneration, including miRNA 4443 (Ge et al., 2022), miR-421, whose downregulation has been shown as protective against cerebral ischemia injury (Yue et al., 2020), and hsa-miRNA-216b-5p which play a role in apoptosis (Dolapçi et al., 2023). Therefore, modulation of these miRNAs might be a new opportunity to avoid RGC degeneration in glaucoma.

This study has some limitations that should be noted. First, the *in vivo* study was performed using a limited number of animals and with only female rats. Thus it could be argued that any non-significant findings were due to the study lacking in power, and any sex differences in the EVs effects have gone unnoticed. Second, optic nerve grading was conducted by three investigators, which despite being blinded, could lead to subjectivity in results. Further studies could be performed by more quantitative methods.

In summary, our study provides the first evidence of EV released from R-28 cells and their neuroprotective effect on RGCs, both *in vitro* and *in vivo*, possibly miRNA-mediated. Our results identify EVs from the R-28 cell line as a promising treatment for RGC degeneration in glaucoma.

## Data Availability

*No additional data are available*.

## References

[R1] Abello J, Nguyen TDT, Marasini R, Aryal S, Weiss ML (2019). Biodistribution of gadolinium- and near infrared-labeled human umbilical cord mesenchymal stromal cell-derived exosomes in tumor bearing mice. Theranostics.

[R2] Arteaga-Blanco LA, Mojoli A, Monteiro RQ, Sandim V, Menna-Barreto RFS, Pereira-Dutra FS, Bozza PT, Resende RO, Bou-Habib DC (2020). Characterization and internalization of small extracellular vesicles released by human primary macrophages derived from circulating monocytes. PLoS One.

[R3] Buckingham BP, Inman DM, Lambert W, Oglesby E, Calkins DJ, Steele MR, Vetter ML, Marsh-Armstrong N, Horner PJ (2008). Progressive ganglion cell degeneration precedes neuronal loss in a mouse model of glaucoma. J Neurosci.

[R4] Chen YH, Huang YC, Chen CH, Wen YT, Tsai RK, Chen C (2023). Investigation of the protective effect of extracellular vesicle miR-124 on retinal ganglion cells using a photolabile paper-based chip. Invest Ophthalmol Vis Sci.

[R5] da Silva-Junior AJ, Mesentier-Louro LA, Nascimento-Dos-Santos G, Teixeira-Pinheiro LC, Vasques JF, Chimeli-Ormonde L, Bodart-Santos V, de Carvalho LRP, Santiago MF, Mendez-Otero R (2021). Human mesenchymal stem cell therapy promotes retinal ganglion cell survival and target reconnection after optic nerve crush in adult rats. Stem Cell Res Ther.

[R6] Dolapçi İB, Noyan S, Polat AY, Gürdal H, Dedeoğlu BG (2023). miR-216b-5p promotes late apoptosis/necroptosis in trastuzumab-resistant SK-BR-3 cells. Turk J Biol.

[R7] Durmaz E, Kutnyanszky M, Mead B (2023). Isolation and culture of primary retinal ganglion cells from rodent retina. Methods Mol Biol.

[R8] Esmaeili M, Mead B (2023). Differentiation of human embryonic/induced-pluripotent stem cells to retinal ganglion cells. Methods Mol Biol.

[R9] Ge X, Yao T, Zhang C, Wang Q, Wang X, Xu LC (2022). Human microRNA-4433 (hsa-miR-4443) targets 18 genes to be a risk factor of neurodegenerative diseases. Curr Alzheimer Res.

[R10] Guo X, Kong X, Huang R, Jin L, Ding X, He M, Liu X, Patel MC, Congdon NG (2014). Effect of Ginkgo biloba on visual field and contrast sensitivity in Chinese patients with normal tension glaucoma: a randomized, crossover clinical trial. Invest Ophthalmol Vis Sci.

[R11] Ito YA, Belforte N, Cueva Vargas JL, Di Polo A (2016). A magnetic microbead occlusion model to induce ocular hypertension-dependent glaucoma in mice. J Vis Exp.

[R12] Kwong JM, Quan A, Kyung H, Piri N, Caprioli J (2011). Quantitative analysis of retinal ganglion cell survival with Rbpms immunolabeling in animal models of optic neuropathies. Invest Ophthalmol Vis Sci.

[R13] Libby RT, Anderson MG, Pang IH, Robinson ZH, Savinova OV, Cosma IM, Snow A, Wilson LA, Smith RS, Clark AF, John SW (2005). Inherited glaucoma in DBA/2J mice: pertinent disease features for studying the neurodegeneration. Vis Neurosci.

[R14] Libby RT, Li Y, Savinova OV, Barter J, Smith RS, Nickells RW, John SW (2005). Susceptibility to neurodegeneration in a glaucoma is modified by Bax gene dosage. PLoS Genet.

[R15] Lorber B, Berry M, Logan A, Tonge D (2002). Effect of lens lesion on neurite outgrowth of retinal ganglion cells in vitro. Mol Cell Neurosci.

[R16] Mathew B, Ravindran S, Liu X, Torres L, Chennakesavalu M, Huang CC, Feng L, Zelka R, Lopez J, Sharma M, Roth S (2019). Mesenchymal stem cell-derived extracellular vesicles and retinal ischemia-reperfusion. Biomaterials.

[R17] Mathew B, Acha LG, Torres LA, Huang CC, Liu A, Kalinin S, Leung K, Dai Y, Feinstein DL, Ravindran S, Roth S (2023). MicroRNA-based engineering of mesenchymal stem cell extracellular vesicles for treatment of retinal ischemic disorders: Engineered extracellular vesiclesand retinal ischemia. Acta Biomater.

[R18] Mead B, Logan A, Berry M, Leadbeater W, Scheven BA (2013). Intravitreally transplanted dental pulp stem cells promote neuroprotection and axon regeneration of retinal ganglion cells after optic nerve injury. Invest Ophthalmol Vis Sci.

[R19] Mead B, Tomarev S (2017). Bone marrow-derived mesenchymal stem cells-derived exosomes promote survival of retinal ganglion cells through miRNA-dependent mechanisms. Stem Cells Transl Med.

[R20] Mead B, Amaral J, Tomarev S (2018). Mesenchymal stem cell-derived small extracellular vesicles promote neuroprotection in rodent models of glaucoma. Invest Ophthalmol Vis Sci.

[R21] Mead B (2023). Microbead-induced ocular hypertension in a rodent model of glaucoma. Methods Mol Biol.

[R22] Miralles de Imperial-Ollero JA, Vidal-Villegas B, Gallego-Ortega A, Nadal-Nicolás FM, Salinas-Navarro M, Norte-Muñoz M, Di Pierdomenico J, Galindo-Romero C, Agudo-Barriuso M, Vidal-Sanz M, Valiente-Soriano FJ (2023). Methods to identify rat and mouse retinal ganglion cells in retinal flat-mounts. Methods Mol Biol.

[R23] Pan D, Chang X, Xu M, Zhang M, Zhang S, Wang Y, Luo X, Xu J, Yang X, Sun X (2019). UMSC-derived exosomes promote retinal ganglion cells survival in a rat model of optic nerve crush. J Chem Neuroanat.

[R24] Rodriguez AR, de Sevilla Müller LP, Brecha NC (2014). The RNA binding protein RBPMS is a selective marker of ganglion cells in the mammalian retina. J Comp Neurol.

[R25] Samsel PA, Kisiswa L, Erichsen JT, Cross SD, Morgan JE (2011). A novel method for the induction of experimental glaucoma using magnetic microspheres. Invest Ophthalmol Vis Sci.

[R26] Seigel GM, Mutchler AL, Imperato EL (1996). Expression of glial markers in a retinal precursor cell line. Mol Vis.

[R27] Seigel GM, Sun W, Wang J, Hershberger DH, Campbell LM, Salvi RJ (2004). Neuronal gene expression and function in the growth-stimulated R28 retinal precursor cell line. Curr Eye Res.

[R28] Seigel GM (2014). Review: R28 retinal precursor cells: the first 20 years. Mol Vis.

[R29] Seigel GM, Yuan K, Goldsmith ZK, Morales-Tirado VM (2018). Heterogeneous R28 retinal precursor cells predominantly express retinal ganglion cell and glial cell markers. Invest Ophthalmol Vis Sci.

[R30] Seyedrazizadeh SZ, Poosti S, Nazari A, Alikhani M, Shekari F, Pakdel F, Shahpasand K, Satarian L, Baharvand H (2020). Extracellular vesicles derived from human ES-MSCs protect retinal ganglion cells and preserve retinal function in a rodent model of optic nerve injury. Stem Cell Res Ther.

[R31] Shinozaki Y, Kashiwagi K, Koizumi S (2023). Astrocyte immune functions and glaucoma. Int J Mol Sci.

[R32] Sluch VM, Chamling X, Liu MM, Berlinicke CA, Cheng J, Mitchell KL, Welsbie DS, Zack DJ (2017). Enhanced stem cell differentiation and immunopurification of genome engineered human retinal ganglion cells. Stem Cells Transl Med.

[R33] Urcola JH, Hernandez M, Vecino E (2006). Three experimental glaucoma models in rats: comparison of the effects of intraocular pressure elevation on retinal ganglion cell size and death. Exp Eye Res.

[R34] Weber AJ, Zelenak D (2011). Experimental glaucoma in the primate induced by latex microspheres. J Neurosci Methods.

[R35] Weinreb RN, Liebmann JM, Cioffi GA, Goldberg I, Brandt JD, Johnson CA, Zangwill LM, Schneider S, Badger H, Bejanian M (2018). Oral memantine for the treatment of glaucoma: design and results of 2 randomized, placebo-controlled, phase 3 studies. Ophthalmology.

[R36] Wiklander OP, Nordin JZ, O’Loughlin A, Gustafsson Y, Corso G, Mäger I, Vader P, Lee Y, Sork H, Seow Y, Heldring N, Alvarez-Erviti L, Smith CI, Le Blanc K, Macchiarini P, Jungebluth P, Wood MJ, Andaloussi SE (2015). Extracellular vesicle in vivo biodistribution is determined by cell source, route of administration and targeting. J Extracell Vesicles.

[R37] Yu F, Wang Y, Huang CQ, Lin SJ, Gao RX, Wu RY (2023). Neuroprotective effect of mesenchymal stem cell-derived extracellular vesicles on optic nerve injury in chronic ocular hypertension. Neural Regen Res.

[R38] Yue Y, Zhao H, Yue Y, Zhang Y, Wei W (2020). Downregulation of microrna-421 relieves cerebral ischemia/reperfusion injuries: involvement of anti-apoptotic and antioxidant activities. Neuromolecular Med.

[R39] Zhu S, Chen L, Wang M, Zhang J, Chen G, Yao Y, Song S, Li T, Xu S, Yu Z, Shen B, Xu D, Chi ZL, Wu W (2023). Schwann cell-derived extracellular vesicles as a potential therapy for retinal ganglion cell degeneration. J Control Release.

